# First Record of Dark‐sided Flycatcher (*Muscicapa sibirica*) for Temperate North America: Intercontinental Vagrancy and Migratory Misorientation

**DOI:** 10.1002/ece3.73445

**Published:** 2026-04-10

**Authors:** Martin B. H. Freeland, Eve Meier, Patricia Lynch, Jon L. Dunn, Peter Pyle, Chris P. Henry, Maya E. Xu, Rodolfo Dirzo

**Affiliations:** ^1^ Department of Biology Stanford University Stanford California USA; ^2^ California Bird Records Committee Western Field Ornithologists San Luis Obispo California USA; ^3^ Santa Clara Valley Bird Alliance Cupertino California USA; ^4^ Institute for Bird Populations Petaluma California USA; ^5^ Department of Earth Systems Science Stanford University Stanford California USA; ^6^ Stanford Woods Institute for the Environment, Stanford Doerr School of Sustainability Stanford University Stanford California USA

**Keywords:** avian vagrancy, long‐distance migration, misorientation, *Muscicapa sibirica*

## Abstract

Birds are among the most mobile organisms on the planet: many species routinely perform long‐distance migrations that require remarkable feats of physical endurance and complex navigation. Rarely, individual long‐distance migrants may make movements in exceptional directions, placing them beyond their species' ordinary distributions and providing the potential for significant ecological developments, such as the colonization of a new region by the vagrant species, the introduction of novel parasites or pathogens, or the dispersal of seeds and fungal spores. We detail the occurrence of a Dark‐sided Flycatcher ( *Muscicapa sibirica* ), a highly migratory passerine species typically found in East Asia, at an urban site in California on 17–19 September 2025, establishing the first record of this species and genus for temperate North America. We age this individual as a first‐fall bird and discuss molt strategies in the genus *Muscicapa*. We identify it as a member of the nominate subspecies, therefore likely originating from highly migratory populations that breed in northeast Asia. We evaluate mechanisms by which this individual may have arrived in California and discuss the concepts of reversal and mirror‐image misorientation, modes of anomalous navigation that may provide explanations for Palearctic‐Nearctic vagrancy in highly migratory Asian passerines. Our observation represents a noteworthy addition to the known avifauna of temperate North America. In documenting this occurrence, we draw attention to the remarkable but little‐studied process of long‐distance vagrancy in migratory birds.

## Graphical abstract

We document the extraordinary occurrence of a Dark‐sided Flycatcher (*Muscicapa sibirica*)—a migratory passerine from East Asia—in California, USA. We discuss the little‐known phenomenon of intercontinental vagrancy in migratory landbirds and examine this case study in detail, providing analysis of the age, molt status, subspecies, and likely origins of this vagrant bird.

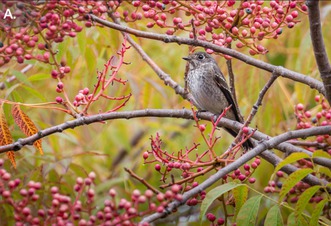

## 1 Introduction

Few regions' avian communities have been so thoroughly inventoried and documented as that of temperate North America (continental North America between the Arctic Circle and the Tropic of Cancer). Additions to the known avifauna of this region are rare, and for the most part consist of remarkable events of long‐distance vagrancy, that is, the movement of an individual bird well beyond the region typically inhabited by the species (Pyle et al. 2015; Chesser et al. 2025). Here, we detail an exceptional instance of intercontinental vagrancy of a Dark‐sided Flycatcher ( *Muscicapa sibirica* )—a species new to the avifauna of temperate North America—occurring in coastal California.

Although avian vagrancy draws great interest among birdwatching hobbyists and has been the subject of some published research, it remains an under‐recognized agent and indicator of ecological change (Dufour, Lees, et al. 2024). Even a single stray individual may be a disperser of zoochorous plants and fungi or a vector of novel pathogens and parasites, and as such facilitate range expansions of other species (Davis and Watson 2018; Ellis et al. 2023). Other ecological effects of vagrancy may be more challenging to predict, as exemplified by the threat of extinction posed to an insular single‐island endemic seabird by predation from vagrant Peregrine Falcons ( *Falco peregrinus* ) (Jiguet et al. 2007). The occurrence of a given vagrant may also be a signal of noteworthy ecological developments in its area of origin, as in, for example, cases of seabirds displaced by El Niño events (England 2000). Furthermore, in certain situations, vagrancy may serve as a mechanism of range expansion and the colonization of new regions by the vagrant species (Veit et al. 2022). The relative importance of these various evolutionary and ecological implications of avian vagrancy remains poorly resolved.

Likewise, much remains to be elucidated regarding not only the consequences, but also the causes of long‐distance vagrancy. A paradigmatic view persists of the typical vagrant individual as a victim of extreme weather or other extrinsic circumstances which cause it to make an extralimital movement. However, this kind of explanation is only capable of accounting for a subset of vagrant records (Howell et al. 2014; Lees and Gilroy 2021), as there remain many that lack any temporal association with storms, strong wind patterns, or other obvious external forces. Certain anomalies in endogenous processes, such as navigation or migratory phenology, may be equally important causes for vagrancy and may occur in individual migrants exhibiting otherwise normal, uninterrupted migratory behavior. Rather than artificial transport or weather‐assisted exogenous vagrancy, in this context we suggest that the concept of reversal misorientation (DeSante 1973)—a form of navigational anomaly in which an individual pursues a route that is a 180° reversal of its ordinary migratory path—may provide a particularly useful framework for the explanation of vagrancy events like that of the Dark‐sided Flycatcher in California.

## 2 Methods

### 2.1 Observation

A small bird of an unknown species was discovered at 10:10 a.m. on September 17, 2025, by coauthors Meier and Lynch at Charleston Marsh, California. They determined that, while resembling a New World flycatcher (Passeriformes: Tyrannidae), the bird's appearance was not consistent with any of the regionally expected flycatchers in the genera *Empidonax* and *Contopus*. At 10:23 a.m. they published a description with low‐resolution photos via eBird (eBird.org, a citizen science database; Sullivan et al. 2009) and through a local email listserv. Shortly after, local birdwatchers including Rachel Lawrence and Jason Altus suggested that the observation might refer to an Old World flycatcher (Passeriformes: Muscicapidae). Other birdwatchers went to search for the bird and Garrett Lau relocated it, obtaining clear photographs at 12:51 p.m. At 1:00 p.m. Steve Rottenborn, an expert in the identification of North American birds, received the photographs and distributed them among other Californian ornithologists via email. Dunn and Freeland learned of the bird in this manner, and Freeland publicized news of the bird's presence on platforms such as the Bay Area Rare Bird Alert beginning at 1:47 p.m. The immediate consensus among the experts consulted by Rottenborn was that Lau's photographs clearly showed an Old World flycatcher of the genus *Muscicapa*, no member of which had previously been recorded in temperate North America, and likely either a Gray‐streaked ( *M. griseisticta* ) or Dark‐sided ( *M. sibirica* ) flycatcher. The announcement of this discovery through eBird and through personal communication attracted hundreds of observers to the location that same afternoon, and hundreds more viewed the bird over the following 2 days. During its stay, the Dark‐sided Flycatcher used an area of approximately two hectares, encompassing willow marsh and an office complex (see below). We observed it feeding at length on the berries of Chinese pistache ( *Pistacia chinensis* ; see Discussion), as well as sallying for insects from the tops of tall trees and perching for extended preening sessions, during which it scratched its head indirectly (i.e., over the wing), as reported for other muscicapids (Gutiérrez‐Ibáñez et al. 2025). It did not vocalize while under observation and did not associate closely with other birds. The bird was last seen at sunset on September 19, despite efforts by many more observers on September 20 and subsequent days.

To supplement our observations of this individual in the field, which were conducted on all 3 days of its presence, we examined the body of reports logged in eBird and iNaturalist (iNaturalist.org, another popular citizen science platform), together with the set of 1032 images of this individual uploaded to eBird and archived in the Macaulay Library (https://www.macaulaylibrary.org/). We also evaluated the 6643 images of other individual Dark‐sided Flycatchers in the Macaulay Library as of 1 January 2026. We further referred to documentation of this individual provided by observers to the California Bird Records Committee (CBRC), an organization sponsored by the Western Field Ornithologists (WFO) for the maintenance of records related to bird species rarely occurring in the state of California. This array of evidence permitted us to assess the bird's age, molt status, and identification at the specific and subspecific levels. To contextualize this record with respect to weather at the site of observation and in the subject bird's hypothesized area of origin, we used weather data maintained by the United States National Oceanic and Atmospheric Administration (National Weather Service 2025).

### 2.2 Site Description

**FIGURE 1:**
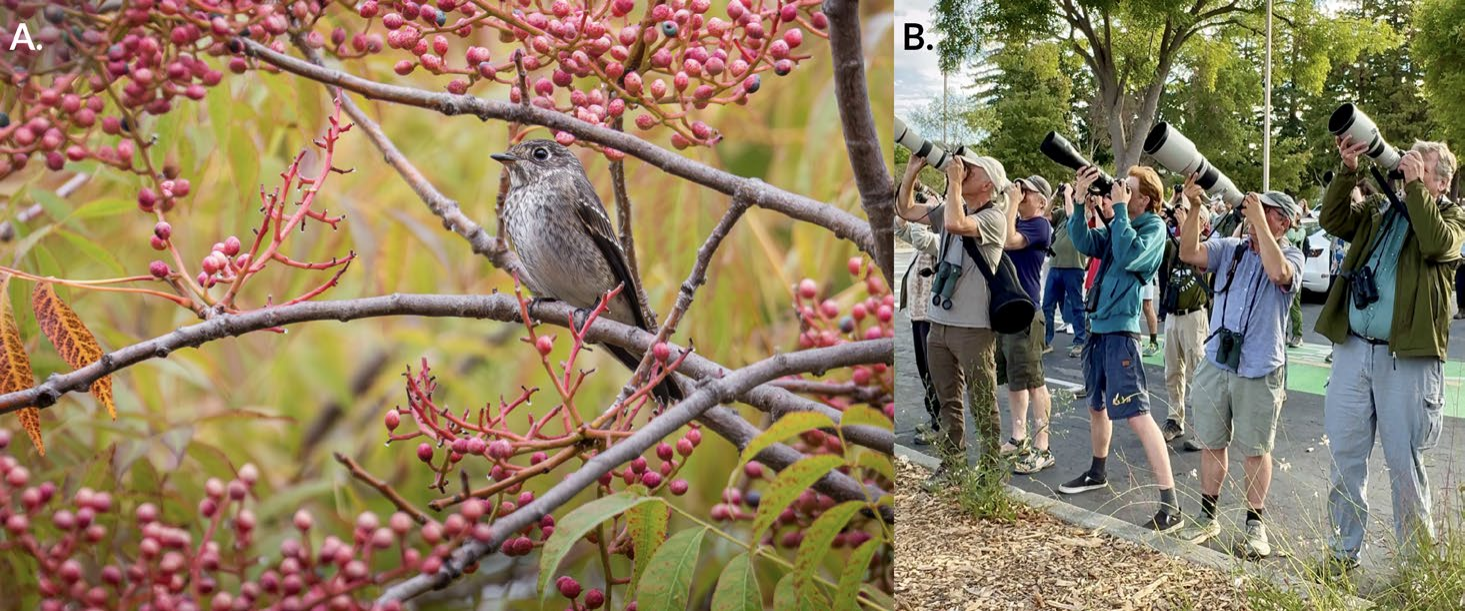
(A) The vagrant Dark‐sided Flycatcher in California, here pictured in a fruiting Chinese pistache tree. It fed extensively on these antioxidant‐rich fruits; this behavior is associated with recovery from or preparation for extreme exertion—such as a long‐distance flight—in other largely insectivorous migratory passerines. (B) Hundreds of birdwatchers converged on the Google campus to view the flycatcher, a first record for temperate North America, over the course of its three‐day stay. Photographs by Michael Long (A) and Chris Henry (B).

The vagrant Dark‐sided Flycatcher was discovered in a small marsh dominated by arroyo willow ( *Salix lasiolepis* ) and cattails (*Typha* sp.) located at the Google corporate headquarters complex in Mountain View, California (37.421, −122.073; 3 MSL). This site is 3 km south of the shore of San Francisco Bay and 32 km east of the Pacific coast at Half Moon Bay. Over the course of its three‐day stay, the flycatcher was observed most frequently in the areas of the Google complex immediately south of the marsh, circling through extensive asphalt parking lots interspersed with landscaping that features ornamental plants. Dominant species include Chinese pistache ( *Pistacia chinensis* ), an exotic tree (Figure 1A), as well as cultivated indigenous species such as coast redwood ( *Sequoia sempervirens* ), giant sequoia ( *Sequoiadendron giganteum* ), sycamores (*Platanus* sp.), toyon ( *Heteromeles arbutifolia* ), blue elder ( *Sambucus cerulea* ), and milkweeds (*Asclepias* spp.). This region of the Google campus has been the subject of extensive revegetation efforts with native plant species reintroduced based on their perceived appeal to birds and certain insects (A. Benham in litt.). This site is characterized by high volumes of pedestrian traffic, particularly so when hundreds of bird enthusiasts converged on the location to view the Dark‐sided Flycatcher (Figure 1B). Remarkably, the first record in the contiguous United States of Siberian Rubythroat (*Calliope calliope*)—another migratory East Asian passerine from the family Muscicapidae—occurred less than 1 km away in November 2022 (Sowa et al. 2023).

## 3 Results

### 3.1 Identification

**FIGURE 2:**
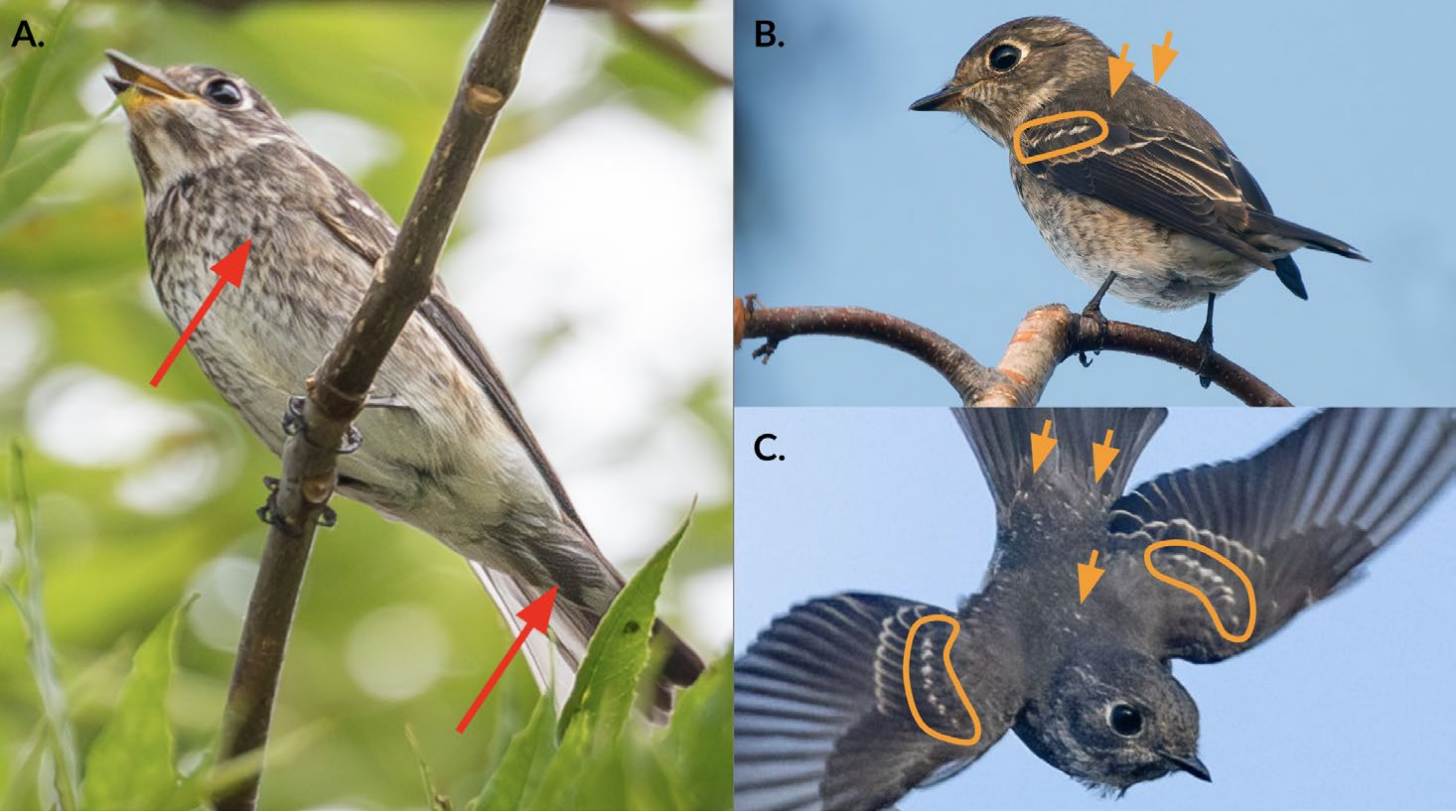
(A) A ventral view of the Dark‐sided Flycatcher shows characteristics diagnostic of this species. The California bird has prominent and extensive dark centers to the undertail covert feathers, which are characteristic of Dark‐sided and do not occur in congeners like Gray‐streaked ( *M. griseisticta* ) and Asian Brown ( *M. dauurica* ) flycatchers; similarly, the chest pattern is unlike the crisp longitudinal gray‐and‐white streaking limited to the sides of the chest seen in Gray‐streaked and equally unlike the diffuse brown wash characteristic of Asian Brown. These features are indicated with red arrows. (B, C) Profile (B) and dorsal (C) views show white‐tipped, juvenile median secondary coverts (orange circles) and white‐tipped body feathers (orange arrows) contrasting with the dark‐tipped, formative feathers surrounding them. The presence of the pale‐tipped juvenile feathers indicates that this is a first‐fall individual. Photographs by Michael Long (A), Mark Chappell (B), and Peter Hart (C).

Many members of the genus *Muscicapa* differ only subtly from one another in morphology. Dark‐sided Flycatcher bears a close resemblance to two congeners in particular: Asian Brown Flycatcher ( *M. dauurica* ) and especially Gray‐streaked Flycatcher. The California bird can be identified as Dark‐sided on the basis of (1) extensive dark centers to the undertail covert feathers (Figure 2A), as the undertail coverts are unmarked white in both other species (Leader 2010) or rarely with limited dark wash at the bases of the feathers in Gray‐streaked, not usually visible in the field and never approaching the extent of darkness visible in the California bird (Alström and Hirschfeld 1991); (2) the pattern of the dark gray‐brown chest markings, which grade from rather diffuse mottling at the sides of the chest to neat dark streaking at the center, and are unlike the sharp gray‐brown vertical stripes limited to the sides of the chest for which Gray‐streaked Flycatcher is named and similarly unlike the uniform brownish wash in that area seen in Asian Brown Flycatcher; (3) the short, stout bill, unlike the slightly longer bill of Gray‐streaked and the even larger, longer bill of Asian Brown; and (4) the length of the primary projection, which exceeds the length of the exposed tertials and is inconsistent both with the somewhat longer primary projection typical of Gray‐streaked and with the much shorter (less than or approximately equal to the length of the exposed tertials) primary projection of Asian Brown (Alström and Hirschfeld 1991; Bradshaw et al. 1991; Leader 2010). The California bird was further distinguished from Asian Brown Flycatcher by (1) the dusky brownish lores and supraloral region, which are plain whitish in Asian Brown; (2) the marked increase (“teardrop”) in the width of the white eyering immediately behind the eye, unlike the uniformly wide eyering of Asian Brown; and (3) the pattern of the throat and upper chest, with strong dark submoustachial and malar stripes separated from the dark markings on the upper chest by neat white bands extending posteriorly from each side of the throat, in contrast to the weaker pattern lacking sharp contrast in this region characteristic of Asian Brown (Clarke et al. 2009; Leader 2010).

Dark‐sided Flycatcher is polytypic and typically treated as including four subspecies: *M. s. sibirica* Gmelin 1789 (including *M. s. opaca* as a junior synonym), *M. s. cacabata* Penard 1919, *M. s. gulmergi* Baker 1923; and *M. s. rothschildi* Baker 1923. These subspecies are divided into two groups, with the monotypic Siberian group including only the nominate subspecies and the Sino‐Himalayan group comprising the three remaining subspecies (Vaurie 1959; Clement 2020a). The three subspecies of the Sino‐Himalayan group are weakly differentiated and intergrade clinally with one another (Vaurie 1959), leading Wells (2007) to suggest that *gulmergi* and *rothschildi* might best be synonymized with *cacabata*. However, the Sino‐Himalayan group differs strongly from nominate *sibirica* and the two groups may warrant recognition as separate species (Clement 2020a).

The nominate subspecies is a long‐distance migrant, breeding from the Russian central Altai northeast to Transbaikalia, east to central Kamchatka, and south to central Japan, and wintering in southeast Asia, largely from the Thai‐Malay Peninsula to Sumatra, West Java, and Borneo (Vaurie 1959; Wells 2007). Migrants in Hong Kong (Carey et al. 2001), much of eastern China (Norevik et al. 2020), and Ko Man Nai in the Gulf of Thailand (P. D. Round in litt.) are referable to this subspecies. In comparison, subspecies of the Sino‐Himalayan group are less migratory, breeding from northwest Pakistan and northeast Afghanistan east through the Himalayas southern Qinghai and Gansu, China, and northern Myanmar and Vietnam, with poorly‐known wintering distributions in southeast Asia (Vaurie 1959; Clement 2020a). Most references (e.g., Vaurie 1959; Clement 2020a) suggest that Dark‐sided Flycatcher winters in southern China, but there are evidently few confirmed records, although fall passage migrants may occur as late as early December (Carey et al. 2001). The winter ranges and precise migratory routes of both subspecies groups require additional study.

The nominate subspecies is separable from the subspecies of the Sino‐Himalayan group on the basis of the extent of the dark markings on the sides: these are much more restricted and sharply‐defined in the nominate subspecies, and the middle of the belly is unmarked white, while the three subspecies of the Sino‐Himalayan group feature much more extensive dark markings on the sides (especially in *M. s. rothschildi*), and show a smaller, slightly brown‐tinted whitish zone in the center of the belly (Vaurie 1959; Svensson 1992; Clement 2020a). Additionally, the nominate subspecies shows a partial white collar and relatively extensive white in the throat, while the Sino‐Himalayan group has a restricted area of white in the throat and typically lacks the white collar (Wells 2007). The California bird displayed limited zones of dark mottling on the sides and flanks, a wide white patch on the belly, and a well‐defined partial white collar, consistent only with the plumage of the nominate subspecies.

### 3.2 Age

The California flycatcher can be aged as a first‐fall bird (i.e., an individual in its first autumn, having hatched during the previous Northern Hemisphere summer) by the white‐tipped median secondary coverts and the presence of multiple contour‐feathers in the mantle and rump with small white tips (Figure 2B,C). No white‐tipped feathers in these tracts are present in definitive plumages, the plumages held by individuals over 1 year of age (Alström and Hirschfeld 1991). Instead, these white‐tipped feathers are juvenile, produced during the prejuvenile (first prebasic) molt in the nest. The shape and quality of the outer primaries and outer rectrices of the California bird—narrow, tapered, and tinged brown in color—are also consistent with the typical appearance of juvenile feathers and not with those of adults (Norevik et al. 2020).

The California bird was not wholly in juvenile plumage; it had already molted most (but not all) body feathers and lesser coverts, those without white tips being replaced by formative feathers. The “post‐juvenile” molt (see below) of a better‐studied congener, the Spotted Flycatcher ( *Muscicapa striata* ), occurs on or near the breeding grounds and is reported to include the upperwing lesser coverts, 1–3 inner median coverts, and some but not all body feathers (Cramp and Perrins 1993; Jenni and Winkler 2020). The extent of feather replacement shown by the California bird is similar to that described for this molt in Spotted Flycatcher, with most body feathers and lesser coverts replaced but most or all median and greater coverts retained.

Notably, the presence of white‐tipped feathers in the mantle and among the upperwing coverts in a first‐year bird that has already commenced fall migration is characteristic of Dark‐sided but not of Gray‐streaked or Asian Brown flycatchers; these last two species often undergo more replacement of juvenile body feathers and wing coverts on summer grounds, resulting in the absence of pale‐tipped juvenile feathers in the dorsum and among the median coverts by the time of the first southbound migratory departure (Leader 2010; Norevik et al. 2020).

Molts away from breeding grounds are, in general, poorly known, and the life‐cycle terminology used by many European ornithologists (e.g., by Cramp and Perrins 1993; Shirihai and Svensson 2018; Jenni and Winkler 2020) can conflate single suspended preformative molts with both “post‐juvenile” (see below) and first prealternate molts (Pyle 2022a). These authors often conclude that the “post‐juvenile” molt occurs only on breeding grounds and that all feather replacement on the winter grounds, whether occurring once or twice in first‐cycle birds, is part of “pre‐breeding” molts. We instead agree with the implications of Leader (2010) and Norevik et al. (2020) that the preformative (“post‐juvenile”) molt of Dark‐sided Flycatcher can be suspended and later complete at stopover locations or upon arrival on the wintering grounds, as occurs during the preformative molt of many North American passerine birds (Pyle 2022b). The body of photographs of Dark‐sided Flycatchers from the species' ordinary range, archived in the Macaulay Library, includes examples of individuals on or close to winter grounds during the first week of November with white‐spotted juvenile feathers that appear to be undergoing or completing preformative molt (e.g., Macaulay Library assets 612914725, 74634671, and 76673761); however, among hundreds of photographs taken in December–May we located none showing retained juvenile feathers. We thus conclude that the Dark‐sided Flycatcher arrived in California following a suspended preformative molt of some but not all body feathers and upperwing coverts, and that some body feathers (with white tips), most or all median coverts, and all greater coverts and flight feathers were juvenile. The preformative molt of this bird, should it survive, typically would complete at a stopover location or on the winter grounds. Based on examination of Macaulay Library images, the preformative molt appears to be complete, as reported for Spotted Flycatcher by Cramp and Perrins (1993) and Jenni and Winkler (2020), although Norevik et al. (2020) imply that it can be partial in some Dark‐sided Flycatchers.

Using Humphrey‐Parkes terminology, we thus consider Dark‐sided Flycatcher to undergo a complete preformative molt that commences with a few feathers on the summer grounds and concludes at stopover sites or (after suspension) on the winter grounds, and complete prebasic molts that occur largely on or near the summer grounds but may be completed at stopover locations. These are followed by partial first and definitive prealternate molts in spring that renew body feathers and sometimes a few proximal greater coverts, as evidenced by molt limits between fresher scapulars and most or all wing feathers on spring birds (e.g., Macaulay Library assets 635662897, 428199831, 560931501; Norevik et al. 2020). Dark‐sided Flycatcher undergoes a distal sequence of primary molt in both the preformative and the prebasic molts, proceeding from the innermost to the outermost primary (e.g., Macaulay Library assets 256493571, 623873079, and 33653041 show distal replacement of primaries during prebasic molt on the summer grounds in Nepal and northern India, and asset 174148081 shows the same pattern in a southbound migrant in Thailand); this is consistent with the pattern reported for an Asian Brown Flycatcher in Thailand (Wells 2007) and differs from the proximal molt replacement sequence (from the outermost primary inwards) reported in Spotted Flycatcher (Cramp and Perrins 1993; Shirihai and Svensson 2018; Jenni and Winkler 2020). Replacement sequences are evolutionarily highly conserved in most birds (Pyle 2013) and the difference in direction of primary replacement among these closely related species is noteworthy.

The sexes cannot readily be distinguished on the basis of plumage in this genus (Norevik et al. 2020).

## 4 Discussion

For this record of a Dark‐sided Flycatcher in coastal California, we found no evidence to suggest the involvement of artificial mechanisms of occurrence, such as escape from captivity or ship assistance. This individual displayed none of the physical or behavioral trademarks of captive origin, such as a leg band, toe amputation, abnormal molt patterns or feather wear, or tameness. Additionally, Dark‐sided Flycatcher, like most other migratory insectivorous passerines, is extremely difficult to maintain in captivity and unknown in North American collections (Bruslund et al. 2025, American Association of Zoos and Aquariums in litt.). On the other hand, the nominate subspecies of Dark‐sided Flycatcher—of which this individual is a member—displays behaviors (e.g., obligate long‐distance migration) and morphological traits (e.g., a long pointed wing, permitting aerodynamically efficient flight) that are positively associated with species‐level propensity to vagrate (Vaurie 1959; Vincze et al. 2019; Sheard et al. 2020; Lees and Gilroy 2021; Brooks 2024). Indeed, Dark‐sided Flycatcher has a track record of natural vagrancy to the Bering Sea region and (once) mainland northern Alaska (Howell et al. 2014; eBird 2025), Iceland and northwestern Europe (Stühmer 2005; Brynjólfsson et al. 2020; eBird 2025), and Bermuda (Wingate 1983). Interestingly, Gray‐streaked Flycatcher, which displays the same behaviors and shares morphological traits associated with potential for vagrancy—notably the very long wings—lacks any substantial pattern of long‐distance extralimital occurrence, never having been recorded more than c. 1500 km out of range (Clement 2020b). The occurrence of the California bird coincided with the ordinary timing of Dark‐sided Flycatcher fall migration (as migrants at approximately the latitude of California in this species' ordinary range occur mainly from late August through early October; Carey et al. 2001, Norevik et al. 2020), and the prevalence of vagrancy in migratory landbirds is highly elevated during fall migration compared with the remainder of the annual cycle. This is in large part due to naïve first‐fall individuals (such as this bird), which are over‐represented as vagrants, often making anomalous movements during their first attempts to migrate (Ralph and Wolfe 2018; Lees and Gilroy 2021; Newton 2023; Dufour, Hellström, et al. 2024). The California Bird Records Committee accepted this record unanimously as relating to a wild bird of natural origin (CBRC #2025‐072).

Dark‐sided Flycatcher is largely insectivorous (Clement 2020a) and rarely documented consuming fruit, but we observed this individual feeding at length on the berries of Chinese pistache (Figure 1A), along with Western Bluebird ( *Sialia mexicana* ), Northern Mockingbird ( *Mimus polyglottos* ), House Finch ( *Haemorhous mexicanus* ), and other local birds. Although these berries sometimes contain insect larvae, the main parasites of this fruit in California are wasps (e.g., the chalcid seed wasp, *Megastigmus pistaciae*) whose larvae are located in the seeds and not the mesocarp (Rejmánek 2014). Therefore, these parasitoids are likely to be excreted along with the remainder of the seed, so characterizing the ingestion of pistache berries as frugivory is still warranted. Many migratory birds, including species that are primarily insectivorous, have been documented preferentially to consume fruits with high concentrations of dietary antioxidants during stopovers on migration and shortly after the completion of migration, likely either to offset prophylactically or repair therapeutically oxidative damage caused by the release of reactive oxygen species during the burning of fat for migratory flights (Bolser et al. 2013; Cooper‐Mullin and McWilliams 2016; McWilliams et al. 2021). Although little information exists relating specifically to the antioxidant content of ornamental cultivars of Chinese pistache, other forms of this species (e.g., *P. c. integerrima*) and other related species (*P. vera, P. lentiscus, P. khinjuk, P. terebinthus, P* *. atlantica* ) store high concentrations of compounds showing strong antioxidant activity in their berries (Belhachat et al. 2017; Noureen et al. 2017; Ahmad et al. 2020; Akyuz et al. 2022; Yuan et al. 2022; Alsharairi 2025). In some cases, fruit consumption may also be used to accumulate fat stores that fuel migratory flights more rapidly than is possible with the consumption of insects alone (Bairlein 1990; Newton 2023). Both the antioxidant and fat‐building roles of fruit consumption in primarily insectivorous birds are principally applicable to individuals that have either just performed, or will shortly perform—or both—a demanding migratory flight. While not definitive, these circumstances are also consistent with the characterization of this individual as a vagrant of natural, unassisted origin.

Although exogenous vagrancy induced by extreme weather is also a form of natural origin, it is unlikely to be the primary mechanism of occurrence in this case because extreme weather conditions did not exist either in northeastern Russia or along the Pacific coast of North America at or just before the time of the Dark‐sided Flycatcher's occurrence. Howell et al. (2014) suggested that the negative phase of the Pacific–North American teleconnection, a large‐scale climate pattern affecting the East Asian jet stream, may help propel Eurasian vagrants such as Dusky Warbler ( *Phylloscopus fuscatus* ) and Red‐throated Pipit ( *Anthus cervinus* ) crossing the Pacific Ocean on a southeasterly heading; however, the teleconnection was in its positive phase over the three days prior to the Dark‐sided Flycatcher's discovery and a very weak negative phase earlier in the month (National Weather Service 2025). At no time in the two weeks preceding the flycatcher's occurrence was the teleconnection likely strong enough to entrain and transport a bird attempting to move in a different direction. Some prior records of Dark‐sided Flycatcher in the Western Hemisphere (e.g., many of the 23 spring records for the Bering and Chukchi sea regions) have been associated with periods of westerly winds that may drift migrants from the Russian Far East toward Alaskan islands (Howell et al. 2014), but the California record was not clearly associated with any such system.

The question then is by what process, outside of captive transportation or entrainment in a weather system, might a Dark‐sided Flycatcher reach temperate North America? A plausible partial explanation for this exceptional record may be given by the concept of reversal misorientation (Nisbet 1962; DeSante 1973; Pyle et al. 2015), which proposes that some individuals of migratory species—especially first‐fall birds—may migrate using a route that is the reverse (i.e., a 180° inversion of the ordinary migratory vector) of the route typically taken by members of their natal population. In most cases, this will lead to vagrancy.

**FIGURE 3:**
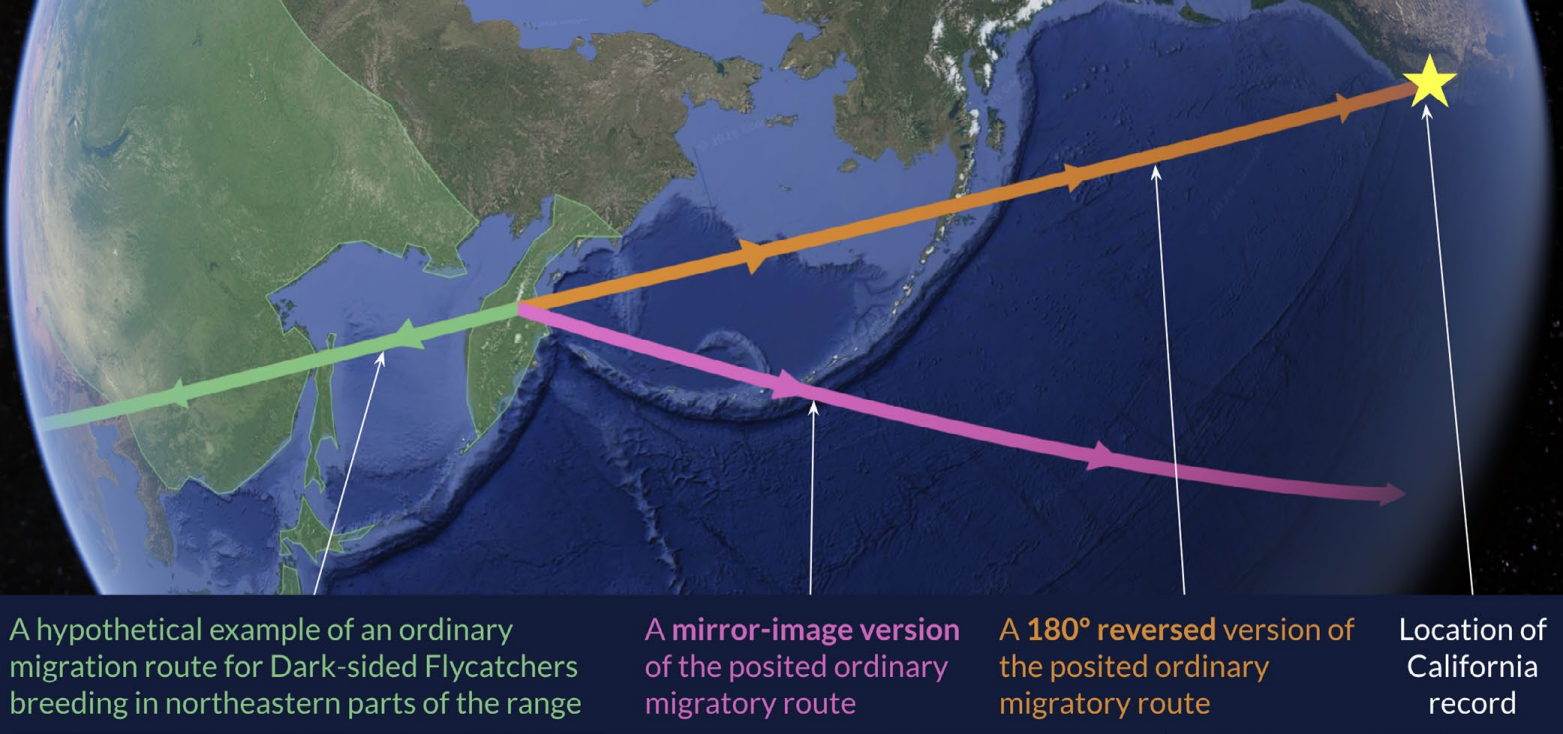
A speculative illustration of how reversal misorientation may have transmuted an ordinary fall migration route into a trajectory bringing a Dark‐sided Flycatcher from the northeastern part of the breeding range (which is shaded in green) to California, while mirror‐image misorientation may be less likely to result in vagrancy to North America. The routes shown lie along Great Circle lines. Imagery 2026 NASA, map data 2026 Google.

A 180° reversal of the ordinary fall migration vector of a Dark‐sided Flycatcher from northeastern breeding populations might place it on a trajectory intersecting the Pacific coast of North America (**Figure** **3**) and central California. The distance traveled along such a path might exceed that traveled along the first leg of an ordinary fall migration route, but the previous occurrence of a vagrant Dark‐sided Flycatcher on Bermuda attests to this species' ability to survive long oceanic crossings, despite the absence of such obstacles from its ordinary migratory routes (Wingate 1983). Mirror‐image misorientation (*sensu* DeSante 1973; Diamond 1982), a different form of abnormal navigation in which an individual follows a migratory route that is a reflection of the typical route across a north–south axis (i.e., a mirror image of its ordinary trajectory), is less likely as an explanation of this individual's occurrence in California. A mirror image of the typical migration vector of a *sibirica* Dark‐sided Flycatcher might be more likely to place vagrants on a trajectory toward the south‐central Pacific Ocean, rather than the North American continent (Figure 3).

Other proposed mechanisms of intercontinental vagrancy include overshoot vagrancy, in which an individual continues along its normal migratory heading beyond the typical distance and thereby moves outside its usual range, and social vagrancy, in which an individual associates closely with heterospecifics and follows the other species' migration route into a novel area. However, California's geographic position renders it effectively unreachable by overshooting fall migrant Dark‐sided Flycatchers, as this species' fall migration should ordinarily take it from northeast Asia to southeast Asia. Social vagrancy is similarly unlikely: no passerine breeding sympatrically with Dark‐sided Flycatcher regularly winters in California or farther south in the New World, muscicapids are not known to migrate socially, and the Dark‐sided Flycatcher in California was not associating closely with other migratory birds.

Not all instances of intercontinental vagrancy fall into these categories, and many cases are not immediately consistent with a single mechanistic framework. However, spatial and temporal patterns consistent with reversal or mirror‐image misorientation during fall migration occur in several East Asian passerines reaching the Western Hemisphere, including other muscicapids such as the Red‐flanked Bluetail ( *Tarsiger cyanurus* )—which has been recorded seven times in California alone—as well as Old World warblers (*Phylloscopus*), wagtails (*Motacilla*), buntings (*Emberiza*), and others (Howell et al. 2014; Lees and Gilroy 2021).

The causes of navigational anomalies such as reversal misorientation remain obscure, and it is conceivable that misorientation, although generally perceived as an endogenous mechanism of vagrancy, may itself be produced by environmental factors. Geomagnetic disturbance (Tonelli et al. 2023), weather in outer space (Gulson‐Castillo et al. 2023), and contamination by certain pollutants (Vyas et al. 1995; Eng et al. 2017) have all been associated with increased rates of vagrancy or navigational abnormality in migratory passerines, perhaps including vagrancy by reversal misorientation. Further research is needed to characterize these pathways in greater detail.

While we lack the ability to provide complete, definitive explanations of the mechanism and causes of the occurrence of a Dark‐sided Flycatcher in temperate North America, our analysis of the record is consistent with its characterization as a scenario of natural, unassisted vagrancy by a first‐fall individual having undergone reversal misorientation. Continued documentation of such occurrences is essential to clarifying the potential ecological and evolutionary ramifications of “black swan events” (Anderson et al. 2017) like intercontinental avian vagrancy. This record adds a new species and genus to the avifauna known to have occurred in temperate North America and provides further demonstration of the remarkable ability of long‐distance migratory birds to make extralimital movements even bypassing biogeographical barriers as formidable as the Earth's widest ocean.

## Author Contributions

**Martin B. H. Freeland:** conceptualization (equal), investigation (equal), project administration (lead), writing – original draft (lead), writing – review and editing (lead). **Eve Meier:** investigation (lead), writing – original draft (supporting), writing – review and editing (supporting). **Patricia Lynch:** investigation (lead), writing – original draft (supporting), writing – review and editing (supporting). **Jon L. Dunn:** conceptualization (equal), investigation (equal), writing – original draft (supporting), writing – review and editing (equal). **Peter Pyle:** conceptualization (supporting), writing – original draft (supporting), writing – review and editing (equal). **Chris P. Henry:** conceptualization (supporting), investigation (equal), writing – review and editing (supporting). **Maya E. Xu:** investigation (equal), writing – review and editing (supporting). **Rodolfo Dirzo:** funding acquisition (lead), supervision (lead), writing – review and editing (supporting).

## Funding

This work was supported by Division of Biological Infrastructure, RCN‐UBE 2216814.

## Conflicts of Interest

The authors declare no conflicts of interest.

## Data Availability

Macaulay Library/eBird photographic data are publicly available from https://search.macaulaylibrary.org/catalog?taxonCode=dasfly&regionCode=US‐CA&sort=rating_rank_desc and iNaturalist data are publicly available from https://www.inaturalist.org/observations?place_id=14&taxon_id=12987. Data from the California Bird Records Committee is publicly accessible (at https://www.californiabirds.org/queryDatabase.asp?species=dark‐sided+flycatcher) in a limited form to protect observer privacy, but may be requested in full from the Secretary of the committee (secretary@californiabirds.org) for research and scholarship.
